# Prognostic significance of cortactin levels in head and neck squamous cell carcinoma: comparison with epidermal growth factor receptor status

**DOI:** 10.1038/sj.bjc.6604245

**Published:** 2008-02-12

**Authors:** P Hofman, C Butori, K Havet, V Hofman, E Selva, N Guevara, J Santini, E Van Obberghen-Schilling

**Affiliations:** 1 Laboratory of Clinical and Experimental Pathology, Pasteur Hospital, Nice, France; 2 INSERM ERI-21, Faculty of Medicine, University of Nice – Sophia Antipolis, Nice, France; 3 Tissue Biobank Unit, Pasteur Hospital, Nice, France; 4 Department of Oto-Rhino-Laryngology, Pasteur Hospital, Nice, France; 5 Institute of Signaling, Developmental Biology and Cancer Research UMR CNRS 6543, Centre Antoine Lacassagne, Nice, France

**Keywords:** cortactin, head and neck squamous cell carcinoma, prognosis, EGF receptor, tissue microarray

## Abstract

Cortactin is an actin-binding Src substrate involved in cell motility and invasion. In this study, we sought to examine the prognostic importance of cortactin protein expression in head and neck squamous cell carcinoma (HNSCC). To do so, cortactin and EGF receptor (EGFR) expression was retrospectively evaluated by immunohistochemistry in a tissue microarray composed of 176 HNSCCs with a mean follow-up time of 5 years. Cortactin immunoreactivity was weak to absent in normal epithelial tissue. Overexpression of the protein in 77 out of 176 tumours (44%) was associated with more advanced tumour-node-metastasis stage and higher histologic grade. Cortactin overexpression was associated with significantly increased local recurrence rates (49 *vs* 28% for high and low expressing carcinomas, respectively), decreased disease-free survival (17 *vs* 61%), and decreased the 5-year overall survival of (21 *vs* 58%), independently of the EGFR status. In multivariate analysis, cortactin expression status remained an independent prognostic factor for local recurrence, disease-free survival, and overall survival. Importantly, we identified a subset of patients with cortactin-overexpressing tumours that displayed low EGFR levels and a survival rate that equalled that of patients with tumoral overexpression of both EGFR and cortactin. These findings identify cortactin as a relevant prognostic marker and may have implications for targeted therapies in patients with HNSCC.

Despite recent advances in cancer treatment, the 5-year overall survival rate for patients with squamous cell carcinoma of the head and neck (HNSCC) has not improved over the last 30 years (Forastiere *et al*, 2001; Greenlee *et al*, 2001). The primary cause of treatment failure in patients with early-stage disease is the development of second primary tumours, whereas patients who present with locally advanced disease are at risk for local–regional recurrence and metastasis, even with the use of near-tolerance doses of radiation (Day and Blot, 1992). Most of the current prognosis factors (such as tumour-node-metastasis (TNM) staging, surgical margin status, and perineural invasion) fail to provide definitive information regarding the biological behaviour of the tumour and its potential to recur. Thus, identifying a biological marker that correlates with recurrence would provide more accurate information on prognosis and enable the treating physician to select a more aggressive course of therapy for high-risk patients. Such a marker could also potentially serve as a target for drugs designed to seek out and exploit specific molecular defects in cancerous cells.

Tumour cell motility is required for local–regional invasion and metastasis. Motility is initiated by protrusion of the leading edge of the cell, resulting in the production of polarised lamellipodia oriented towards the direction of movement (Yamaguchi and Condeelis, 2007). Cortactin is a ubiquitously expressed Src substrate that engages in several protein–protein interactions that may be functionally relevant to tumoral progression. It accumulates in lamellipodia of motile cells where it plays a crucial role in the dynamic regulation of the actin cytoskeleton by virtue of its binding to filamentous (F) actin, Arp2/3, and components of the cortical actin-polymerising machinery (Weed and Parsons, 2001; Daly, 2004). Cortactin also colocalises with F-actin in invadopodia of metastatic tumour cells, where it participates in degradation of the extracellular matrix around tumours (Artym *et al*, 2006; Yamaguchi *et al*, 2006). The cortactin gene *CTTN* (formerly *EMS1*) resides on chromosome locus 11q13 that is frequently amplified in human cancers, including over 30% HNSCCs (Brookes *et al*, 1993; Schuuring, 1995; Huang *et al*, 2006; Gibcus *et al*, 2007). Thus, the functional properties of cortactin, together with amplification of chromosome locus 11q13, suggest a link between its overexpression and tumour invasion and/or metastasis. Accordingly, in several animal models ectopic overexpression of cortactin has been shown to accelerate tumour dissemination, and decreased expression of cortactin, by RNA interference, leads to impaired tumour cell migration and metastasis (Li *et al*, 2001; Chuma *et al*, 2004; Hill *et al*, 2006; Luo *et al*, 2006).

A growing list of cortactin-binding partners has been identified, including proteins that regulate receptor clustering, endocytosis, and vesicle transport such as dynamin 2 (McNiven *et al*, 2000) and CD2AP (CIN85-related protein) (Lynch *et al*, 2003). Recent results from studies in cultured cells (Lynch *et al*, 2003; Timpson *et al*, 2005) indicate that cortactin could participate in receptor-mediated endocytosis of the epidermal growth factor (EGF) receptor (EGFR). Thus, cortactin overexpression attenuated ligand-induced downregulation of EGFR and led to sustained activation of EGFR signalling. Conversely, reduction of cortactin expression in an 11q13-amplified HNSCC cell line by RNA interference accelerated EGFR degradation (Timpson *et al*, 2005). On the basis of these findings, it was proposed that overexpression of cortactin may contribute to increased EGFR expression and signalling in malignancies with *CTTN* amplification. This is of particular importance in HNSCC, since enhanced EGFR signalling/expression has been associated with aggressive disease and poor prognosis (Dassonville *et al*, 1993; Grandis and Tweardy, 1993; Kalyankrishna and Grandis, 2006) and EGFR-targeted molecular therapeutics are currently being examined in clinical settings (for recent reviews, see Astsaturov *et al*, 2006a, 2006b and Reuter *et al*, 2007).

To determine whether cortactin expression could be an independent prognostic factor in HNSCC, we have investigated expression of the protein in a large series of HNSCCs using tissue microarray (TMA) technology and have correlated this expression to the outcome of these patients. Epidermal growth factor receptor staining was performed to investigate the potential linkage between cortactin and EGFR overexpression.

## MATERIALS AND METHODS

### Patient selection

Surgically removed tumours embedded in paraffin wax blocks from 176 cases of HNSCCs were retrieved from the archives of the Department of Pathology (Pasteur Hospital) at the University of Nice, Nice, France. The cases, received between 1993 and 2001, included squamous cell carcinomas exclusively from patients without metastases; basaloid type carcinoma and other nonmucosal tumours were excluded. Cases were selected to build TMAs and included in this study only if a follow-up of at least 5 years was obtained, and clinical data were available. Mean age at surgery was 63 years (range: 37–82) and 138 patients were male. Selected patients displayed similar health status and absence of concurrent chronic illnesses, or tumours elsewhere. The primary sites of the carcinomas were oral cavity (*n*=40), pharynx (*n*=57; including oropharynx, *n*=31 and hypopharynx, *n*=26), and larynx (*n*=79). Tumour size according to the TNM classification was T1 (*n*=34), T2 (*n*=44), T3 (*n*=48), and T4 (*n*=50). Eighty-seven per cent of the carcinomas were keratinising, with a range of grades: grade I (*n*=27), grade II (*n*=94), and grade III (*n*=55).

### Tissue microarray construction

Tissue microarray construction and high-throughput analysis of tissue samples were performed in accordance with Institutional Guidelines. Haematoxylin and eosin-stained sections of primary tumours were reviewed, and areas of tumour and normal tissue were marked on the slides. Areas of necrosis and keratin pools were avoided. Representative carcinoma areas were selected for building TMAs and arrays were designed as previously described (Hoos and Cordon-Cardo, 2001). Briefly, three tissue cores (600 *μ*m in diameter) corresponding to three representative central areas of the tumour were selected. The tissue cores were arrayed into a recipient paraffin block using a fine steel needle and an automatic tissue microarrayer (Beecher Instruments, Sun Prairie, WI, USA and Alphelys, Paris, France). Tissue microarrays of primary carcinomas contained normal tissue adjacent to benign oral tumours from patients (six tissue cores from biopsies performed on these patients), who served as control and for regulating mark spacing between core centres; cores were spaced at intervals of 1 mm in the *x* and *y* axes. A 4-*μ*m haematoxylin- and eosin-stained section was reviewed to confirm the presence in TMAs of morphologically representative areas of the original lesions.

### Immunohistochemistry

Immunohistochemical methods were performed on serial 2 *μ*m deparaffinized TMA sections. For cortactin detection, intrinsic peroxidase was blocked by incubating sections with 3% hydrogen peroxide for 6 min. Sections were blocked in 4% goat serum for 20 min, then incubated for 30 min with mouse monoclonal anticortactin antibody (clone 4F11; Upstate, Lake Placid, NY, USA and Euromedex, Souffelweyersheim, France). After rinsing with PBS, sections were incubated with biotinylated secondary antibody for 20 min, rinsed with PBS, and incubated with anti-mouse Ig's streptavidin complexed with biotinylated peroxidase (StreptABComplex/HRP; DAKO Corp., Carpinteria, CA, USA) for 20 min. Sections were then washed with distilled water, incubated with developing solution (3-amino-9-ethylcarbazole; DAKO), counterstained with haematoxylin, and mounted with aqueous mounting medium. Epidermal growth factor receptor was detected on sequential sections using the Invitrogen immunodetection kit (mouse anti-EGFR clone 31G7), as indicated by the manufacturer. After staining, slides were analysed with an image-analysis workstation (Spot Browser version 7; Alphelys), as previously described (Pages *et al*, 2005). Signal intensity of digitised images was determined using the Spot Browser software; grey values (0=black to 255=white) ranged between 102 and 142, and a value inferior or equal to 120 was defined as overexpression. In parallel, staining of the cores was scored by pathologists based on signal intensity (0–3) and the percentage of positive cells (0⩽10%, 1=10–25%, 2=25–50%, and 3⩾50%). Overexpression of cortactin and EGFR was arbitrarily defined as more than 50% of cells strongly (score 3) stained. Input from visual inspection data and detection of events in digitised images was stored in dedicated tables for comparison and statistical analysis. Discrepancies were resolved by three pathologists (CB, VH, and PH) using a multihead microscope. A disc was considered unsuitable for analysis if it was completely absent, it contained no tumour tissue (sampling error), or it contained too few tumour cells (<10%) for analysis (uninformative). For each patient, the mean score of a minimum of two core biopsies was calculated. Whole-tissue sections from tumour blocks of a subset of 30 cases were stained and compared by visual inspection with the corresponding TMA discs using the above-mentioned scoring criteria. These cases were selected to include 10 cases of each grade, and clinicopathological data of these cases were very similar to those of the whole series (not shown). Negative controls for staining used in this study were normal oral mucosa for cortactin and normal striated muscle for EGFR.

For figures, polychromatic high-resolution spot images (1392 × 1040 pixels) were obtained using a Leica DMR optic microscope (Leica Microsystems, Rueil-Malmaison, France) equipped with a CoolSNAP EZ cooled charge-coupled device camera (Roper Scientifique, Evry, France).

### Statistics

Histospots containing <10% of a tumour were excluded from additional analyses. Disease-free survival, overall survival, and local recurrence were assessed by Kaplan–Meier analysis with log-rank score for determining statistical significance. All survival analyses were performed at 5-year cutoffs. Relative risk was assessed by the univariate and multivariate Cox proportional hazards model. Comparison of cortactin overexpression with the clinical and pathological variables including gender, TNM stage, histological grade, and tumour type (primary *vs* recurrent) was made using *χ*^2^ analysis. Calculations and analyses were performed with SPSS 11.5 for Windows (SPSS Inc., Chicago, IL, USA) and where appropriate, were two tailed.

Nonparametric correlation (Spearman's *ρ* coefficient; SPSS release 12.01) was used to compare data from whole-tissue sections and discs; and to relate the mean scores obtained from four discs to the scores obtained from individual discs, the mean scores of two discs, and the mean scores for three discs. The degree of agreement between data from whole-tissue sections and the mean of four discs was assessed using weighted Cohen's *κ* coefficient (SAS for Windows, release 8.02). For descriptive purposes, *κ*<0.4 represents poor-to-fair agreement, 0.4–0.6 moderate agreement.

## RESULTS

### Clinical and pathological variable analysis

One hundred and seventy-six patients who underwent primary surgical resection for HNSCC in the Department of Otorhinolaryngology (CHU of Nice, France) met inclusion criteria to build TMAs. Demographic and clinicopathological variables for the cohort are summarised in Table 1.

### Immunohistochemical analysis of cortactin and EGFR expression

**Figure 1:**
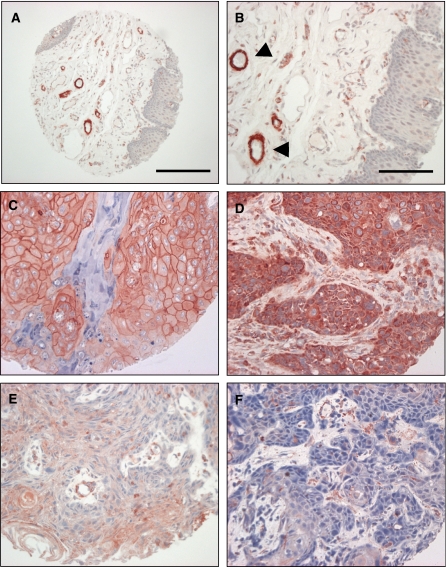
Cortactin expression in normal oral mucosa and HNSCC. Staining of cortactin was performed as indicated in Materials and Methods. Normal epithelium (**A**–**B**) is devoid of staining, arrowheads indicate cortactin staining of blood vessels. Scale bar represents 200 *μ*m in the tissue core shown in panel A and 100 *μ*m in the higher magnification images shown in panels B–F. (**C**–**D**) Squamous cell carcinoma demonstrating strong membrane and cytoplasmic staining, respectively. (**E**) Squamous cell carcinoma exhibiting weak staining. (**F**) Squamous cell carcinoma displaying absence of immunohistochemical staining. Only strong staining (**C**–**D**) was scored as cortactin overexpression. Weak staining or absence of staining (**E**–**F**) was scored as negative for this study. Calibration bar represents 200 *μ*m in images on the left.

Cortactin immunoreactivity in normal epithelial tissue was low to undetectable (Figure 1A–B). Staining was observed in blood vessels of the underlying connective tissue, both in capillary endothelial cells and in *α*-actin-positive smooth muscle cells of larger vessels. Interestingly, the presence of cortactin in endothelial cells and in perivascular cells was often mutually excusive (data not shown). Epidermal growth factor receptor staining in normal epithelium was intense in the basal and suprabasal proliferative layers and undetectable in the connective tissue and blood vessels (not shown).

Of the HNSCC tumours that met inclusion criteria, all were interpretable for cortactin staining. Tumours displayed one of the three distinct immunostaining phenotypes (Figure 1): intense diffuse membranous and/or cytoplasmic staining (Figure 1C–D), weak cytoplasmic and/or membranous staining (Figure 1E), and absence of staining (Figure 1F). Cortactin overexpression was defined as intense diffuse cytoplasmic and/or plasma membrane staining in >50% of tumour cells. In all, 77 out of 176 tumours were designated as cortactin overexpressors. No staining was observed in any spots stained with the negative control reagent. Comparisons were made between clinical/pathological variables and cortactin status by *χ*^2^ analysis. Cortactin overexpression was significantly associated with higher TNM stage (*P*=0.005), as 38 out of the 77 cortactin-overexpressing tumours (49%) were stage IV, as compared with 50 out of the total 176 tumours analysed (28%). Cortactin overexpression was also associated with higher histologic grade as 44 out of 77 cortactin-overexpressing tumours were poorly differentiated (*P*=0.001). There was no association between cortactin status and the other clinical/pathological variables indicated in Table 1. In particular, no association between cortactin status and primary tumour site (oral cavity, oropharynx or hypopharynx, and larynx) was noted.

**Figure 2:**
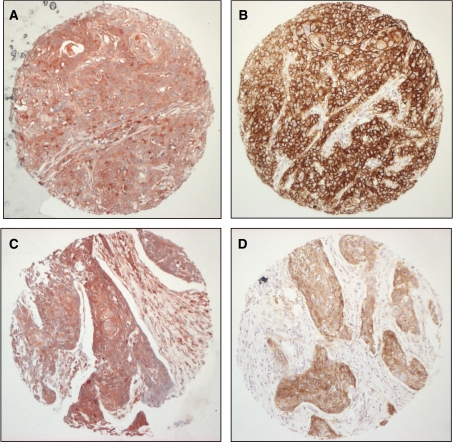
Epidermal growth factor receptor and cortactin expression in HNSCC. Squamous cell carcinoma demonstrating both strong cortactin (**A**) and EGFR (**B**) coexpression. Squamous cell carcinoma demonstrating cortactin overexpression (**C**) and EGFR nonoverexpression (**D**). (Scale bar=200 *μ*m).

Epidermal growth factor receptor overexpression is known to be associated with advanced TNM stage, higher histologic grade, and recurrent head and neck tumours (see Dei Tos and Ellis, 2005). This was confirmed for the present cohort by analysis of EGFR expression, as summarised in Table 1. To examine the possible association between the pattern of EGFR expression and cortactin status, a pairwise analysis was performed. Interestingly, none of the tumour specimens with low cortactin levels (cortactin nonoverexpressors) overexpressed the EGFR. Tumours that overexpress cortactin were largely positive for EGFR (Figure 2A–B). However, in our series we identified a group of 29 tumours with intense cortactin expression in which the EGFR was not overexpressed (Figure 2C–D). These include 10 T1, 9 T2, and 9 T3 (all N0M0) tumours. Ten of these tumours were well differentiated and 19 tumours were moderately or poorly differentiated.

Importantly, cortactin and EGFR expression levels observed in the TMA discs faithfully reflected the staining intensity of these proteins in whole-tissue sections from corresponding tumour blocks in a subset of 30 cases (see Materials and Methods section).

### Survival analysis

#### Local recurrence

**Figure 3:**
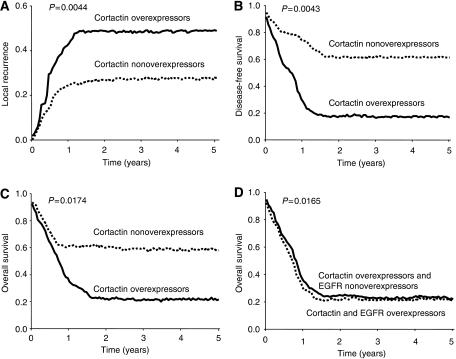
Kaplan–Meier estimates of the 5-year local recurrence (**A**), disease-free survival (**B**), and overall survival (**C**), by cortactin status. Cortactin overexpressors had significantly lower disease-free and lower survival rates than nonoverexpressors. Additionally, cortactin overexpressors had higher local recurrence rates than nonoverexpressors. Kaplan–Meier estimates of the 5-year overall survival by both EGFR and cortactin status (**D**).

The expression status of cortactin, as determined by immunohistochemistry, was evaluated for association with local recurrence using Kaplan–Meier survival analysis with log-rank statistics for determining significance. This analysis demonstrated that cortactin overexpression is associated with increased local recurrence rates. Patients with cortactin nonoverexpression have a local recurrence rate of 28%, compared with 49% for cortactin-overexpressing patients (Figure 3A). Among these latter patients with cortactin-overexpressing tumours, no differences relating to the initial treatment (postoperative radiotherapy alone or not, and postoperative chemotherapy plus radiotherapy or not) were noted. Univariate Cox analysis revealed a hazard ratio for cortactin overexpressors of 6.0 (*P*=0.014).

### Disease-free survival

Kaplan–Meier survival analysis (Figure 3B) demonstrated that cortactin overexpression is associated with decreased disease-free survival. Patients with cortactin nonoverexpression have a 5-year disease-free survival of 61%, compared with 17% for cortactin overexpression (*P*=0.0040). Univariate Cox analysis revealed a hazard ratio for cortactin-overexpressing patients of 2.6 (*P*=0.007).

### Overall survival

Kaplan–Meier survival analysis (Figure 3C) demonstrated that cortactin overexpression is associated with decreased overall survival. Patients without cortactin overexpression had a 5-year overall survival of 58%, compared with 21% for cortactin overexpression (*P*=0.0170). Univariate Cox analysis revealed a hazard ratio for cortactin-overexpressing patients of 2.3 (*P*=0.024).

Results for univariate survival analysis of local recurrence, disease-free survival, and overall survival are summarised in Table 2. In parallel, the expression status of EGFR was evaluated for association with overall survival. As mentioned above, EGFR overexpression is associated with decreased overall survival. Results for univariate survival analysis of local recurrence, disease-free survival, and overall survival for this cohort are summarised in Table 3. Interestingly, patients with tumours that do not overexpress the EGFR but overexpress cortactin had a 5-year overall survival of 22%, similar to patients with tumours that overexpress both EGFR and cortactin (Figure 3D).

### Multivariate analysis

Using the Cox proportional hazards model, we performed multivariate analysis to assess the independent predictive value of cortactin expression for 5-year disease-free survival, overall survival, and local recurrence. The following prognostic variables were also included tumour type (primary *vs* recurrent), TNM stage, and histologic grade. For disease-free survival, only cortactin expression status remained an independent prognostic factor (*P*=0.01). For overall survival, both cortactin status and TNM stage were independent prognostic factors (*P*=0.038 and 0.014, respectively). The results for the multivariate survival analysis are summarised in Table 2. Similarly, for overall survival both EGFR status and TNM stage were independent prognostic factors (*P*=0.041 and 0.011, respectively). The results for the multivariate survival analysis for EGFR are summarised in Table 3.

## DISCUSSION

Amplification of the *CTTN* gene has been linked to poor prognosis in HNSCC (Rodrigo *et al*, 2000), yet the relationship between cortactin protein levels and prognosis has not been thoroughly investigated to date. Tissue microarrays have previously been shown to represent reliable tools for the identification of cellular and molecular alterations in several tumour types, including HNSCCs (Gomaa *et al*, 2005). Recently, in a small number of HNSCCs, 11 out of 39 (28%) cases, cortactin overexpression determined by immunohistochemistry correlated with lymph node metastasis and *CTTN* gene amplification (Rothschild *et al*, 2006). In the present study of 176 tumours, we found by quantitative immunohistochemical analysis of TMAs, that cortactin expression status in the tumours of patients with HNSCC is a strong independent prognostic factor for local recurrence as well as disease-free and overall survival. Hence, patients with cortactin-overexpressing tumours displayed a five-fold increase in the risk of local recurrence and nearly a three-fold increase in risk of death by any cause. Importantly, cortactin expression status remained an independent prognostic factor in multivariate analysis.

Several gain- and loss-of-function studies over the past few years have convincingly demonstrated the role of cortactin in regulation of cell motility and invasion by virtue of its role in actin remodelling, invadopodia formation, adhesion, endocytosis, and regulation of cell–cell junctions (reviewed in Daly, 2004). Although the acquisition of a motile phenotype has conventionally been regarded as a late event in tumour progression, it was recently proposed that aberrant cell motility may, in addition to being essential to tumour invasion and metastatic dissemination, also contributes significantly to rapid tumour growth (Norton and Massague, 2006). Indeed, cortactin overexpression in our study was associated advanced TNM stage and high histologic grade. From the pathologist's point of view, cortactin overexpression could be a new prognosis marker for HNSCC.

Cortactin is a substrate of c-Src, and activation of Src family tyrosine kinases is known to be critically involved in carcinoma cell migration extracellular matrix degradation and invasive behaviour. In HNSCC cells, c-Src was proposed to be a downstream target of the EGFR, as treatment of cells with the EGFR inhibitor ZD1839 was found to block activation of the c-Src pathway (Yang *et al*, 2004). Src family kinases were also shown to be involved in mediating activation of STATs 3 and 5 in concert with the EGFR in HNSCC cells (Xi *et al*, 2003). This evidence for a functional association between EGFR and Src/cortactin pathways prompted us to examine EGFR levels in our TMAs. In the majority of cases, EGFR overexpression mirrored cortactin overexpression. However, we identified a subset of 29 tumours in which cortactin and EGFR overexpression could be uncoupled. In these tumours, intense cortactin staining occurred in the absence of strong EGFR staining. Interestingly, patients harbouring these tumours displayed a 5-year overall survival rate that was similar to that of patients with tumours that overexpress both EGFR and cortactin (22 *vs* 19%, respectively). This finding was unexpected in light of recent studies by Timpson *et al* (2005) suggesting that cortactin overexpression may contribute to EGFR overexpression. In cultured cells, cortactin overexpression inhibited ligand-induced downregulation of the EGFR (Timpson *et al*, 2005). Although elevated EGFR expression in tumours is usually associated with more aggressive disease and poor clinical prognosis; in some studies EGFR expression was found to have no prognostic significance (see Dei Tos and Ellis, 2005). This may be the case for the subset of cortactin-overexpressing/EGFR-nonoverexpressing tumours that we describe in which activation or overexpression of signalling components downstream of the receptor could influence cell survival and growth. Whereas these patients may not benefit from EGFR-targeting strategies, they could respond to alternate inhibitors such as Src family kinases that are currently undergoing phase I/II evaluations in advanced solid tumours.

Therapies that target the EGFR have generated high hopes for the management of head and neck cancer. Several clinical trials that explore the use of agents directed against the EGFR, alone or together with various combinations of radiation and chemotherapy, and are currently underway. However, as witnessed in various tumour settings, use of such agents is not without undesirable consequences, and treatment can lead to toxicity and acquired drug resistance. Recently, expression of a tumour-specific constitutively active spliced variant of the receptor (EGFRvIII) was detected in 44% of a small cohort of HNSCCs and found to contribute to enhanced growth and resistance to wild-type EGFR-targeting antibodies (Sok *et al*, 2006). Altogether these events highlight the therapeutic advantage that targeting multiple components of the EGFR/Src/cortactin axis could represent. Interestingly, recent studies in mammary tumour cells by Hashimoto *et al* (2006) illustrate the role of cortactin itself as a potential therapeutic target. Thus, blocking the interaction of the cortactin SH3 domain with AMAP1 (a GTP-Arf6 effector overexpressed in invasive mammary tumours) with a cell-permeable peptide derived from the AMAP1 sequence, or a small-molecule compound, was found to effectively inhibit AMAP1/cortactin binding, and breast cancer invasion and metastasis (Hashimoto *et al*, 2006).

Numerous investigations have addressed genomic alterations that accompany HNSCC using various techniques (e.g., comparative genomic hybridisation, DNA microarrays, and fluorescence *in situ* hybridisation). Amplification of the 11q13 locus, where *CTTN* resides, is the most frequent amplification event in HNSCC and may be driven by a set of genes that could cooperatively provide growth or metastatic advantage to cancer cells including *CCND1, FGF4*, *FGF3*, *SHANK2*, and *PAK1* (see Huang *et al*, 2002, 2006 and Gibcus *et al*, 2007). Indeed, recurrent coamplification of the cytoskeleton-associated genes *CCTN* and *SHANK2* with the cell-cycle control gene *CCND1* has been observed in oral squamous cell carcinoma (Freier *et al*, 2006). Future studies are warranted to determine the possible involvement of other gene products of the 11q13 amplicon in HNSCC. For example, both activity and expression of the serine/threonine kinase Pak1, an effector of the Rac and Cdc42 GTPases and potential cortactin accomplice, was found to be elevated in head and neck tumours as compared with adjacent normal tissue biopsy specimens (Yang *et al*, 2004), although this study was limited to a small number of tumours.

In conclusion, high levels of cortactin protein expression in HNSCC were closely associated with poor prognosis. Although the precise mechanism remains to be elucidated, this finding is important for several reasons. First, it identifies cortactin as a strong, independent prognostic indicator in patients with HNSCC. Second, it identifies the Src substrate, cortactin as a relevant prognostic marker and pertinent molecular target for development of new antitumoral agents and raises the possibility that cortactin status may guide clinicians in tailoring future therapy.

## Figures and Tables

**Table 1:** Comparison of cortactin and EGFR status to demographic, clinical, and pathologic data

	**Cortactin status^a^**				**EGFR status^a^**			
	***n***	−	+	***P***	***n***	−	+	***P***
*Patient cohort*								
*Age (years) 37–82 (median 63)*								
Interpretable for cortactin	176	99	77					
Interpretable for EGFR					176	66	110	
								
*Positive control*								
Interpretable for cortactin	7	0	7					
Noninterpretable	1							
Interpretable for EGFR					9	2	7	
Noninterpretable					2			
								
*Gender*								
Male	138	76	62	0.11	138	36	102	0.13
Female	38	23	15		38	30	8	
								
*Site*								
Oral cavity	40	20	20	0.42	40	10	30	0.39
Pharynx	57	42	15		57	21	36	0.11
Larynx	79	66	13		79	35	39	
								
*TNM stage*								
I	34	15	19	0.005^†^	34	24	10	0.004^†^
II	44	19	25		44	20	24	
III	48	15	23		48	16	32	
IV	50	12	38		50	14	36	
								
*Grade*								
Well differentiated	27	15	12	0.001^†^	27	16	11	0.001^†^
Moderately differentiated	94	24	70		94	34	60	
Poorly differentiated	55	11	44		55	11	44	

EGFR=epidermal growth factor receptor; TNM=tumour node metastasis.

a Cortactin/EGFR status: (−)=nonoverexpressor; (+)=overexpressor.

†Significant at the 0.01 level.

**Table 2:** Univariate and multivariate Cox regression analyses by cortactin expression levels

**Cortactin status**	**Mean time to recurrence (months)**	**Percentage of recurrence (95% confidence interval)**	**Hazard ratio (95% confidence interval)**	***P***
*Univariate*				
*Local recurrence*				
Overexpressor	32.0	49.1% (39–66)	6.032 (1.6–27.1)	0.014
Nonoverexpressor	47.0	28.0% (16–49)	0.169 (0.05–0.8)	
				
*Disease-free survival*				
Overexpressor	22.2	61.5% (40–81)	2.586 (1.2–5.9)	0.007
Nonoverexpressor	34.7	17.5% (11–29)	0.379 (0.3–0.8)	
				
*Overall survival*				
Overexpressor	29.2	57.8% (35–80)	2.275 (1.2–4.7)	0.024
Nonoverexpressor	42.5	20.9% (11–30)	0.425 (0.3–0.9)	
				
*Multivariate*				
**Variable**	**Hazard ratio**	**95**% **confidence interval**		***P***
*Local recurrence*				
Tumour type	1.675	0.8–3.7	—	0.20
TNM stage	1.412	0.8–2.4	—	0.20
Histologic grade	0.965	0.6–1.6	—	0.90
Cortactin overexpressor	5.293	1.2–23.6	—	0.029^*^
				
*Disease-free survival*				
Tumour type	1.616	0.9–3.0	—	0.13
TNM stage	1.453	1.0–2.1	—	0.06
Histologic grade	1.033	0.7–1.5	—	0.87
Cortactin overexpressor	3.000	1.3–7.0	—	0.010^*^
				
*Overall survival*				
Tumour type	1.648	0.9–3.1	—	0.12
TNM stage	1.679	1.1–2.5	—	0.014^*^
Histologic grade	0.984	0.7–1.5	—	0.94
Cortactin overexpressor	2.449	1.1–5.7	—	0.038^*^

TNM=tumour node metastasis.

^*^Significant at the 0.05 level.

**Table 3:** Univariate and multivariate Cox regression analyses by EGFR expression levels

**EGFR status**	**Mean time to recurrence (months)**	**Percentage of recurrence (95**% **confidence interval)**	**Hazard ratio (95**% **confidence interval)**	***P***
*Univariate*				
*Local recurrence*				
Overexpressor	35.0	48.1% (38–64)	6.112 (1.3–22.1)	0.011
Nonoverexpressor	49.0	34.0% (30–46)	0.135 (0.03–0.5)	
				
*Disease-free survival*				
Overexpressor	24.2	59.2% (40–79)	2.786 (1.3–5.8)	0.005
Nonoverexpressor	36.7	16% (11–29)	0.323 (0.3–0.6)	
				
*Overall survival*				
Overexpressor	26.2	55.5% (34–79)	2.550 (1.2–4.7)	0.023
Nonoverexpressor	39.5	19.8% (14–30)	0.398 (0.2–0.8)	
				
*Multivariate*				
**Variable**	**Hazard ratio**	**95**% **confidence interval**		***P***
*Local recurrence*				
Tumour type	1.556	0.7–3.5	—	0.25
TNM stage	1.398	0.7–2.6	—	0.25
Histologic grade	0.889	0.5–1.3	—	0.80
EGFR overexpressor	5.377	1.1–20.5	—	0.025^*^
				
*Disease-free survival*				
Tumour type	1.557	0.8–2.8	—	0.11
TNM stage	1.559	1.1–2.5	—	0.08
Histologic grade	1.110	0.9–1.9	—	0.79
EGFR overexpressor	2.599	1.2–6.1	—	0.012^*^
				
*Overall survival*				
Tumour type	1.559	0.8–3.5	—	0.15
TNM stage	1.450	1.3–2.7	—	0.011^*^
Histologic grade	0.999	0.9–1.8	—	0.84
EGFR overexpressor	2.568	1.2–5.9	—	0.041^*^

EGFR=epidermal growth factor receptor; TNM=tumour node metastasis.

^*^Significant at the 0.05 level.
